# Buserelin treatment to rats causes enteric neurodegeneration with moderate effects on CRF-immunoreactive neurons and *Enterobacteriaceae* in colon, and in acetylcholine-mediated permeability in ileum

**DOI:** 10.1186/s13104-015-1800-x

**Published:** 2015-12-28

**Authors:** Elin Sand, Caroline Linninge, Liudmyla Lozinska, Emil Egecioglu, Bodil Roth, Göran Molin, Björn Weström, Eva Ekblad, Bodil Ohlsson

**Affiliations:** Division of Internal Medicine, Department of Clinical Sciences, Skåne University Hospital, Lund University, Inga Marie Nilssons street 32, 205 02 Malmö, Sweden; Neurogastroenterology Unit, Department of Experimental Medical Science, BMC B11, Lund University, 221 84 Lund, Sweden; Department of Food Technology, Engineering and Nutrition, Lund University, 22100 Lund, Sweden; Department of Biology, Functional Biology, Lund University, 221 84 Lund, Sweden; Department of Clinical Neuroscience and Rehabilitation, University of Gothenburg, 405 30 Gothenburg, Sweden

**Keywords:** Corticotropin-releasing factor (CRF), Enteric neuropathy, Gonadotropin-releasing hormone (GnRH), Gut microbiota, Intestinal permeability, Stress responses

## Abstract

**Background:**

The gonadotropin-releasing hormone (GnRH) analog buserelin causes enteric neuronal loss. Acute stress or injection of corticotropin-releasing factor (CRF) affects motility, secretion, and barrier function of the gastrointestinal tract. The aim of the study was to characterize the CRF immunoreactivity in enteric neurons after buserelin treatment, and to evaluate possible effects of enteric neuropathy on gut microbiota, intestinal permeability, and stress response behavior.

**Results:**

Sixty rats were given buserelin (20 μg) or saline subcutaneously for 5 days, repeated four times with 3 weeks in-between. At the study end, enteric neuronal density, enteric expression of CRF, gut microbial composition, and plasma levels of adrenocorticotropic hormone (ACTH) and CRF were analyzed. Intestinal permeability was examined in Ussing chambers and the reaction to stressful events was measured by behavior tests. Buserelin treatment reduced the number of neurons along the entire gastrointestinal tract, with increased relative numbers of CRF-immunoreactive submucosal and myenteric neurons in colon (p < 0.05 and p < 0.01, respectively). The overall microbial diversity and relative abundance did not differ between groups, but *Enterobacteriaceae* was decreased in colon in buserelin-treated rats (p = 0.020). Basal intestinal permeability did not differ between groups, whereas carbachol stimulation increased ileum permeability in controls (p < 0.05), but not in buserelin-treated rats. Buserelin did not affect stress behavior.

**Conclusions:**

Although buserelin treatment leads to enteric neuronal loss along the gastrointestinal tract with an increased percentage of CRF-immunoreactive neurons in colon, the physiology is well preserved, with modest effects on colon microbiota and absence of carbachol-induced permeability in ileum as the only observed changes.

**Electronic supplementary material:**

The online version of this article (doi:10.1186/s13104-015-1800-x) contains supplementary material, which is available to authorized users.

## Background

Gonadotropin-releasing hormone (GnRH) stimulates secretion of follicle-stimulating hormone (FSH) and luteinizing hormone (LH) from the anterior pituitary, with subsequent secretion of steroid hormones from the gonads [1]. Studies indicate a role for the reproductive peptide hormones also in the gastrointestinal (GI) tract, since GnRH immunoreactivity is found in human enteric neurons [2, 3], and LH receptors are expressed in both human and rat myenteric neurons [4–6]. Some women develop severe dysmotility or abdominal pain after treatment with GnRH analogs [2, 3, 7]. In rat, around 50 % of enteric neurons throughout the GI tract were lost after four treatment sessions with the GnRH analog buserelin, whereas morphological measurements showed normal histology in mucosa, submucosa, and muscle layers [5]. Still, the GI function regarding transit time and nutrient absorption was well preserved [8]. If translated into the clinical setting, it may suggest that patients with diffuse GI complaints can suffer from mild to moderate enteric neuropathy despite normal findings in standardized clinical examinations.

Patients with dysmotility and GI symptoms show comorbidity with affective disorders, and their symptoms are influenced by psychological stress [9]. Corticotropin-releasing factor (CRF) is a non-selective ligand to CRF receptor 1 and 2, although preferring CRF receptor 1, and is the principal regulator of pituitary adrenocorticotropic hormone (ACTH) and adrenal glucocorticoid secretion in response to stressful stimuli [10]. Elevated central CRF levels are involved in the etiology of stress-related psychiatric, physiological, and behavioral disorders [11]. Both experimental trials on animals, and clinical studies on man, demonstrate that CRF is implicated in anxiety and depression [12, 13]. CRF and CRF receptor 1 are to a great extent present in the enteric nervous system (ENS), where CRF is expressed in vasoactive intestinal peptide-immunoreactive (VIP-IR) neurons [14–17]. Central or peripheral administration of CRF and endogenously stress-induced CRF release affect motility, secretion, inflammation, and permeability of the GI tract, and CRF added in cell cultures reverse the neuroprotective effects evoked by VIP, whereas administration of CRF antagonists alleviate the effects [10, 14–17]. However, the effect of intestinal CRF on stress behavior and gut function has not been studied.

Stress induces increased permeability of the GI mucosa, thereafter bacteria and luminal factors may cross the epithelial barrier and activate a mucosal immune response. This response may in turn alter the microbiome composition and enhance the hypothalamic–pituitary–adrenal (HPA) drive [18]. The gut microbiota release transmitter-like agents, which communicate with the ENS, and lipopolysaccharides (LPS), which affect the enteric neurons via toll-like receptor-4 (TLR-4) [19]. Gut microbiota may influence the development of psychiatric disorders, since antibodies against LPS from commensal gut microbiota have been found in patients with depression or chronic fatigue syndrome [19]. Thus, both CRF and gut microbiota could play important roles in the development of depression and anxiogenic behaviors.

Since buserelin treatment in man may cause severe GI side effects [2, 3, 7], there is a need to further characterize the rat model of buserelin-induced enteric neuropathy. Furthermore, functional consequences of enteric neuropathy must be studied in a rat model, to learn how to find and diagnose corresponding damage in patients with diffuse GI dysfunction. The aims of the present study were thus to characterize CRF immunoreactivity in enteric neurons in the experimental rat model of repeated treatment with the GnRH analog buserelin, and to evaluate possible effects evoked by enteric neuropathy on gut microbiota, intestinal permeability, and stress response behavior.

## Results

### General observations

**Fig. 1:**
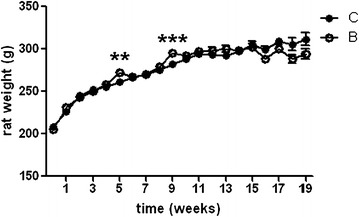
Body weight over time studied on rats treated with one to four sessions with saline (*C*, *unfilled circle*) or buserelin (*B*, *filled circle*). One session consists of 5 days of treatment, with one daily subcutaneous injection of saline or buserelin, followed by 3 weeks of recovery. All rats were healthy and gained weight throughout the experimentation. At the end of the second and third treatments (weeks 5 and 9), buserelin-treated rats showed a transient increase in weight compared with saline-treated rats. Results are presented as means and standard error of the mean (SEM) and analyzed by Student's t test,* C* = 8–24 and* B* = 12–36. Statistical significance is indicated by **p < 0.01 and ***p < 0.001

All animals exhibited normal activity and looked healthy in gross observation throughout the study period, why all individuals were included in the study. At the end of the second and third treatment period (weeks 5 and 9), buserelin-treated rats showed a transient increase in body weight compared with control rats, with no difference in body weight gain between the two groups at sacrifice (Fig. 1). At week 5, the weight gain was 2.3 ± 0.8 % for controls and 5.4 ± 0.8 % for buserelin-treated rats (p < 0.01). At week 9, the weight gain was 2.5 ± 0.6 % and 5.7 ± 0.5 % for controls and buserelin-treated, respectively (p < 0.001). At autopsy, the visceral organs were inspected and no lesions or abnormalities were identified.

### Plasma analyses

Buserelin treatment (B) did not affect the plasma levels of ACTH compared with controls (C) (C = 693 (597–757) pg/ml, B = 694 (456–780) pg/ml). CRF levels in plasma were below detectable values in all rats (<0.039 ng/ml).

### Neuronal survival

Submucosal and myenteric neurons stained well in all rats, irrespective of treatment.

**Fig. 2:**
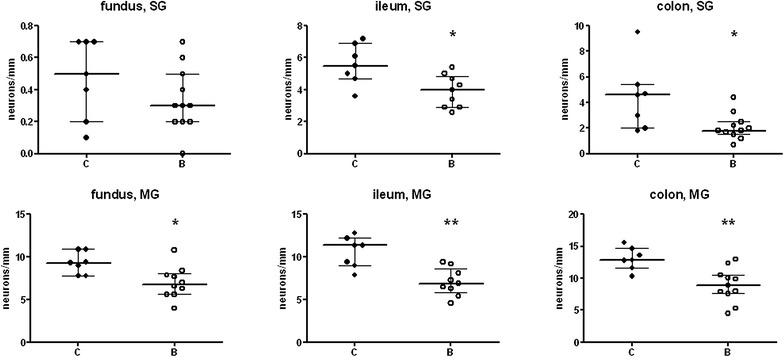
Numbers of neurons in submucosal (SG) and myenteric ganglia (MG) per mm length in fundus, ileum, and colon from rats treated with four sessions of saline (controls, *C*) or buserelin (*B*). Rats were euthanized 2 weeks after the fourth treatment session. Neuronal counting was performed on longitudinally cut, whole-wall sections. Rats subjected to four sessions of buserelin treatment showed decreased numbers of submucosal neurons in ileum (p < 0.05) and colon (p < 0.05), and myenteric neurons in fundus (p < 0.05), ileum (p < 0.01), and colon (p < 0.01), compared with controls. *C* = 7 rats and *B* = 11 rats. Results are presented as medians and interquartile ranges and were analyzed by the Mann–Whitney U-test. Statistical significance is indicated by *p < 0.05 and **p < 0.01

After buserelin treatment, the absolute numbers of submucosal neurons were reduced in ileum and colon, but not in fundus (Fig. 2), whereas the numbers of myenteric neurons were reduced in fundus, ileum, and colon (Fig. 2).

### CRF-immunoreactive neurons

In the fundic part of the stomach, no CRF-IR submucosal neurons were found, whereas a few short fibers were found innervating the mucosa, submucosa, submucosal ganglia, and blood vessel, in both controls and buserelin-treated rats. Around 1–2 % of the myenteric neurons were IR to CRF. Very few fibers innervating the muscle layers and the myenteric ganglia were identified in both groups.

In ileum, 8 % of the submucosal neurons were IR to CRF in controls compared with 13 % in the buserelin-treated group (p = 0.110). Of the myenteric neurons, there was a tendency to increased relative number of CRF-IR neurons in buserelin-treated rats (8 %) compared with controls (5 %) (p = 0.076). Moderate numbers of CRF-IR fibers were seen in the mucosa, submucosal and myenteric ganglia, and around blood vessels, whereas a few of these fibers were seen in the submucosa and muscle layers in both controls and buserelin-treated rats.

**Fig. 3:**
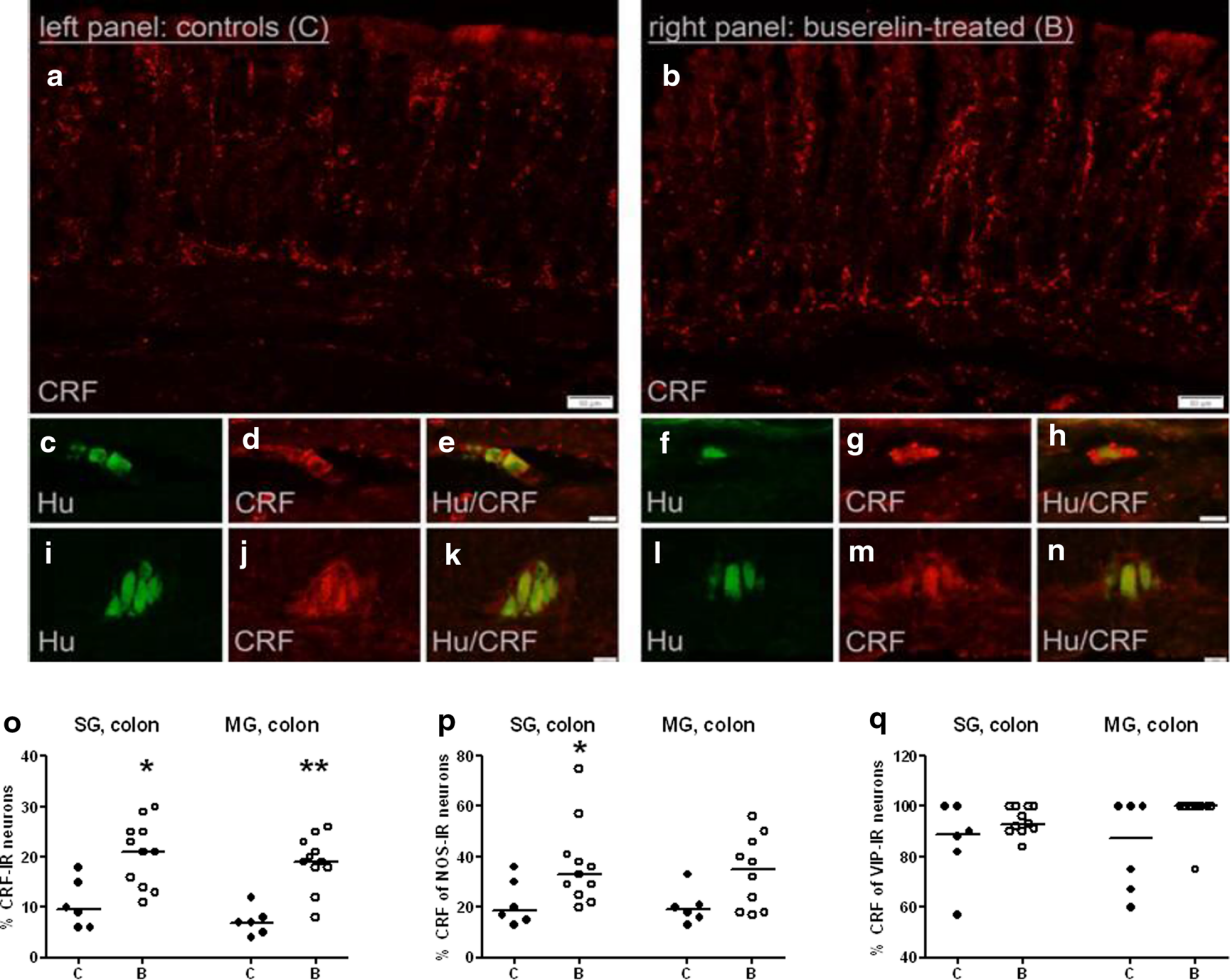
Cryo sections of colon from rats treated with four sessions of saline (controls,* C*) in the *left panel* or buserelin (*B*) in the* right panel*. Double-immunostaining with human neuronal protein (Hu; *green*) and corticotropin-releasing factor (CRF; *red*). Micrographs a and b show intense CRF-immunostaining of fibers in the colon mucosa and submucosa. Micrographs **c**–**h** of submucosal ganglia (SG) are visualizing HuC/D-immunoreactive (IR) neurons (**c**, **f**) also IR to CRF (**d**, **g**). Micrographs** c** and** d** are merged in** e**, and micrographs** f** and** g** are merged in** h**. Micrographs **i**–**n** of myenteric ganglia (MG) are visualizing HuC/D-IR neurons (**i**, **l**) also IR to CRF (**j**, **m**). Micrographs **i** and **j** are merged in **k**, and micrographs **l** and **m** are merged in **n**. In micrograph o, the relative numbers of CRF-IR neurons in SG and MG of colon are presented. The percentage of CRF-IR neurons of the neurons IR to nitric oxide synthase (NOS, **p**) and vasoactive intestinal peptide (VIP, **q**) is presented. Four sessions of buserelin treatment increased the expression of submucosal NOS-IR neurons also showing CRF immunoreactivity (p < 0.05) (**p**), and there was a trend to increased numbers of myenteric NOS- and VIP-IR neurons also IR to CRF, compared with controls (both, p = 0.070) (**p**, **q**).* C* = 5 rats and* B* = 11 rats. Results are presented as individual values and medians and were analyzed by the Mann–Whitney U-test. Statistical significance is indicated by *p < 0.05 and **p < 0.01

In colon, numerous CRF-IR fibers were present. Moderate numbers of CRF-fibers were found in the submucosal and myenteric ganglia, whereas few of these fibers were found in the submucosa, muscle layers, and around blood vessels in both groups of rats (Fig. 3a–b). The relative numbers of CRF-IR neurons in submucosal ganglia were 10 % in controls and 21 % in buserelin-treated rats (p < 0.05) (Fig. 3c–h, o). Seven percent of the myenteric neurons were CRF-IR in controls, which increased to 19 % after buserelin treatment (p < 0.01) (Fig. 3i–n, o). The absolute number of CRF-IR submucosal neurons/mm ganglia in the colon were equal between controls and buserelin-treated rats, whereas the absolute number of CRF-IR myenteric neurons/mm ganglia was increased after buserelin treatment (Additional file 1: Figure S1).

### NOS-immunoreactive neurons in colon

The relative numbers of nitric oxide synthase (NOS)-IR neurons were 30 % in submucosal ganglia and 33 % in myenteric ganglia in both controls and buserelin-treated rats. NOS-IR nerve fibers were abundant in the circular muscle layer and myenteric ganglia. A moderate number of NOS-IR nerve fibers was seen in the submucosal ganglia, whereas few NOS-IR nerve fibers were seen in the mucosa, submucosa, and longitudinal muscle layer. NOS-IR nerve fibers were rarely found around blood vessels. Buserelin treatment did not affect the distribution or staining intensity of NOS-IR neurons.

### VIP-immunoreactive neurons in colon

The relative numbers of VIP-IR neurons were high in submucosal ganglia (24 %) and low in myenteric ganglia (1 %) in both groups. VIP-IR nerve fibers were abundant in the mucosa, submucosa, and submucosal ganglia, moderate in numbers in the circular muscle layer and myenteric ganglia, and few in the longitudinal muscle layer. VIP-IR nerve fibers were detected around the blood vessels. Buserelin treatment did not affect the frequency of VIP-IR nerve fibers in the various layers.

### Co-expression of neuropeptides in colon

Four sessions of buserelin treatment increased the relative number of submucosal NOS-IR neurons also IR to CRF (Fig. 3p), and there was a trend to increased relative numbers of myenteric NOS-IR neurons also IR to CRF, and myenteric VIP-IR neurons also IR to CRF, compared with controls (both, p = 0.070) (Fig. 3p, q).

### Gut microbial composition

#### Diversity of the gut bacterial flora

The diversity of the dominating taxa of the gut microbiota and diversity indices did not differ between control rats and buserelin-treated rats (Additional file 2: Table S2). The overall gut microbial community structure was relatively similar, since no significant differences were observed between the groups when both richness and evenness were considered.

**Fig. 4:**
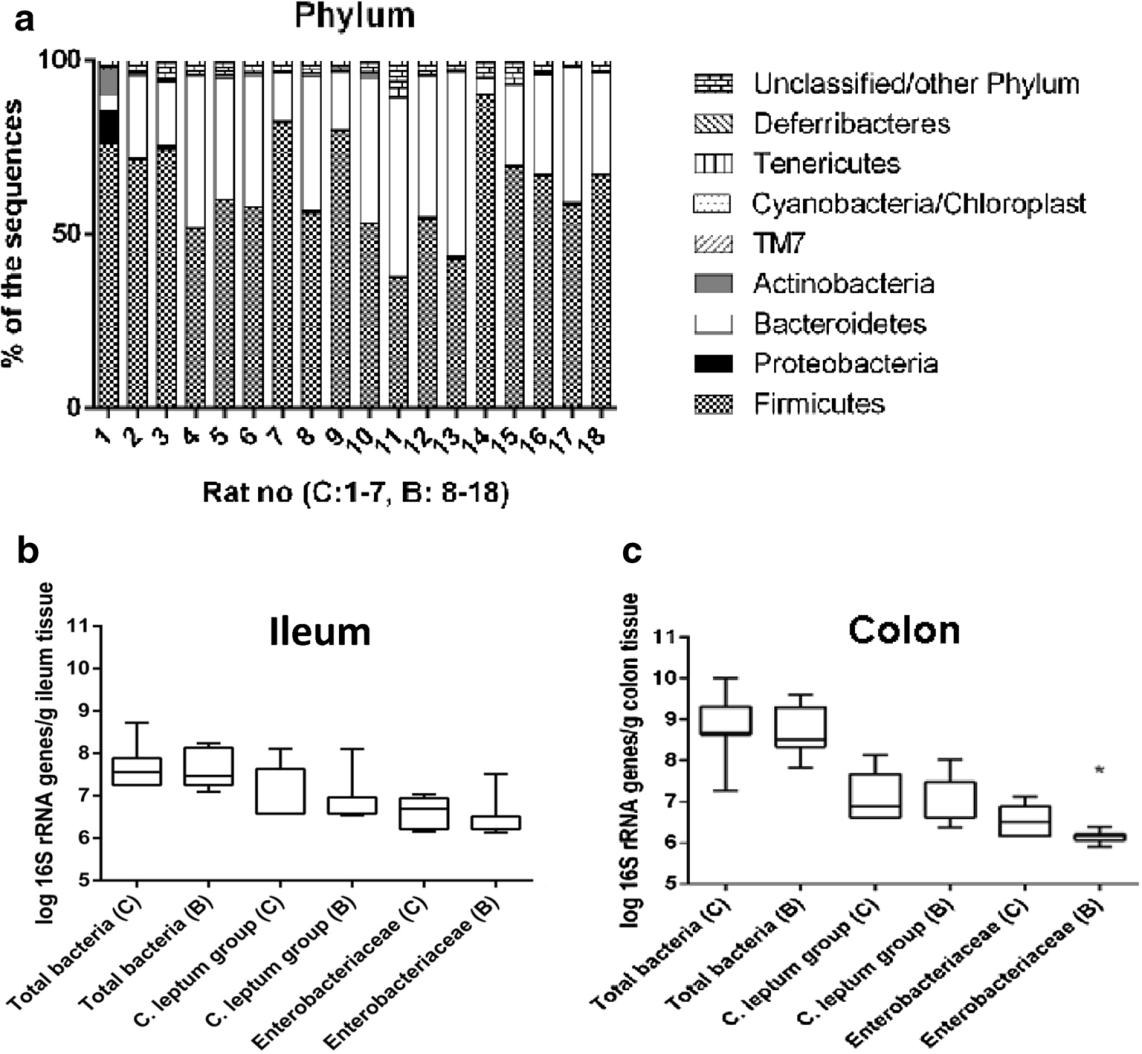
The colonic microbiota was analyzed with next generation sequencing using the ion torrent platform. Firmicutes and Bacteroidetes were the two most dominant phyla in both controls (*C*) and buserelin (*B*)-treated rats. There were no significant differences between the *C* and *B* group regarding the relative abundance of any phyla or families (**a**). PCR-quantified amounts of selected bacterial families in tissue from ileum (**b**) and colon (**c**) of controls and buserelin-treated rats. The amounts of *16S rRNA* gene copies from total bacteria and the *C. leptum* group were not statistically different between the *C* and *B* group in neither ileum nor colon. The amount of *16S rRNA* gene copies from *Enterobacteriaceae* was significantly lower in tissue from colon (p = 0.020), but not ileum, in buserelin-treated rats compared with control rats. Samples under the detection limit were set to the highest possible detection limit. *C* = 7 rats and *B* = 11 rats. Results are presented as medians and interquartile ranges and were analyzed by the Mann–Whitney U-test. Statistical significance is indicated by *p < 0.05

Firmicutes and Bacteroidetes were the two most dominant phyla in both controls and buserelin-treated rats, with no significant differences regarding the relative abundance of any phyla or families between groups (Fig. 4a).

#### Quantification of the gut bacterial flora

In ileum and colon, the total amount of bacteria and the amount of bacteria from the *C.leptum* group did not differ between the two treatments (Fig. 4b, c). The amount of *Enterobacteriaceae* was lower in colonic tissue of rats treated with buserelin compared with the control group (p < 0.05) (Fig. 4c), but significant differences could not be observed in ileum (Fig. 4b). Bacteria from the *B.fragilis* group were not detected in any of the samples in the present study.

#### Intestinal permeability

**Fig. 5:**
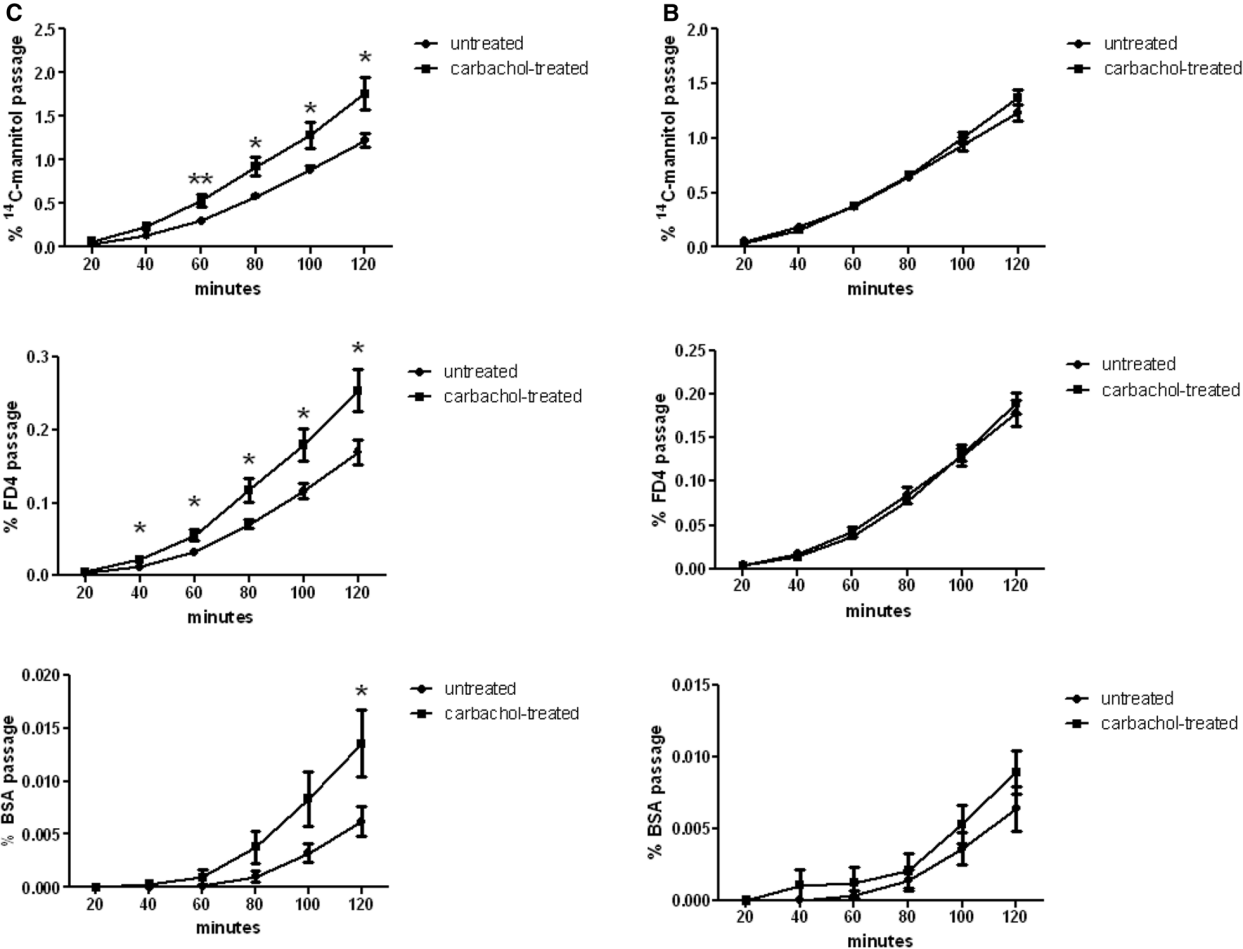
Intestinal permeability studied in ileum of controls (*C*, *left panel*) and buserelin (*B*, *right panel*)-treated rats. Buserelin treatment did not affect baseline permeability in the ileum. Carbachol treatment of ileum in controls significantly enhanced the permeability of d-(1-^14^C)-mannitol (2.9 kBq/mL), fluorescein isothiocyanate (FITC)-dextran (FD4) (1 mg/mL), and at 120 min also bovine serum albumin (BSA) (25 mg/mL), compared with the untreated ileum. Carbachol treatment (10 mM) failed to enhance the permeability of ileum from buserelin-treated rats. *C* = 7 rats and *B* = 11 rats. Results are presented as medians and interquartile ranges and were analyzed by the Mann–Whitney U-test. Statistical significance is indicated by *p < 0.05 and **p < 0.01

During basal conditions, the intestinal permeability of mannitol, fluorescein isothiocyanate (FITC)-dextran, and bovine serum albumin (BSA) in ileum and colon was similar in buserelin-treated rats and control rats (Fig. 5). When stimulating the ileum with carbachol, the permeability of mannitol, FITC-dextran, and BSA (at the latest time-point) increased in control rats (Fig. 5C). In buserelin-treated rats, no increase in permeability due to carbachol stimulation could be detected (Fig. 5B).

#### Stress behavior

**Fig. 6:**
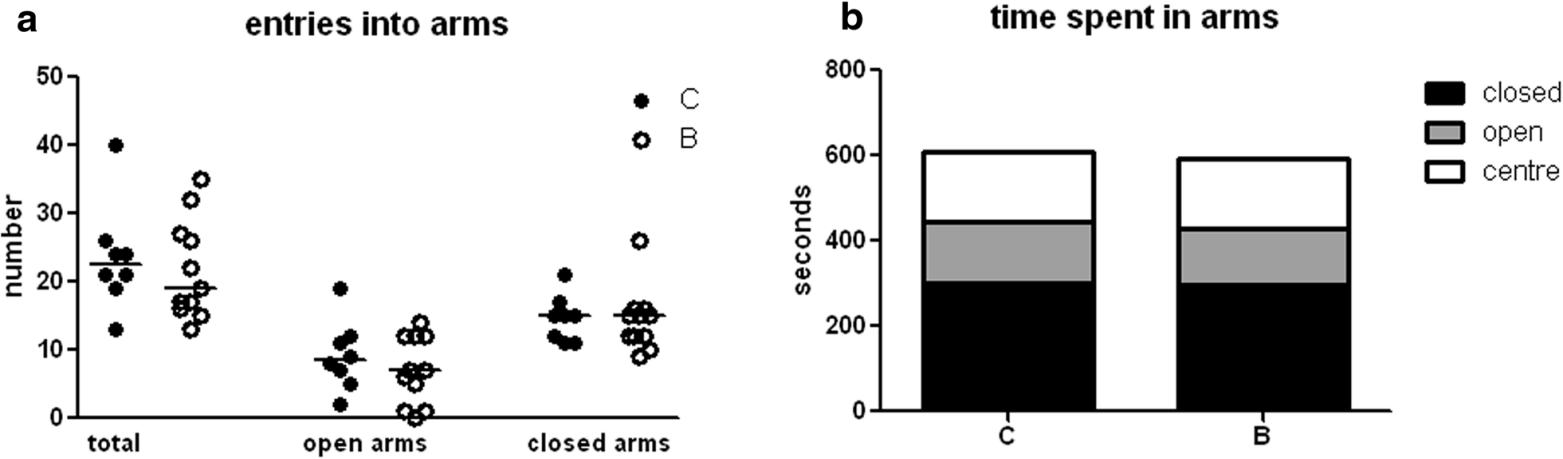
In the elevated plus maze test (EPMT), no differences were detected in the total numbers of entries and numbers of entries into open arms or closed arms (**a**) in the buserelin-treated rats (*B*) compared with controls (*C*). Neither did the time spent in closed arms, open arms or in the center of the platform differ between the two groups (**b**). *C* = 8 rats and *B* = 11 rats. Results are presented as individual values and medians (**a**) or as medians (**b**) and were analyzed by the Mann–Whitney U-test

In the elevated plus maze test (EPMT), the total numbers of entries, or entries into closed or open arms, did not differ between controls and buserelin-treated rats (Fig. 6a). Neither did the percentage of open or closed arm entries differ between the groups (data not shown). The total time spent into closed, open arms or the center platform did not differ between groups (Fig. 6b). There was no difference in how much time controls and buserelin-treated rats spent in the outer parts of the open arms (C = 72 (56–124) s, B = 76 (0–104) s). Neither was there any difference in how many times the rats entered the outer parts of the open arms (data not shown). Open arm head dips or protected arm head dips did not differ between the groups (data not shown). All parameters are presented for the entire 0–10-min period studied. When the data were analyzed in periods of 0–5 and 5–10 min, the results did not differ from the presented results (data not shown).

**Fig. 7:**
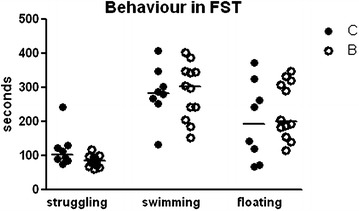
Behaviors are presented in the forced swimming test (FST) as the time of struggling, swimming or floating during a 10-min session. No differences could be found in buserelin-treated rats (*B*) compared with controls (*C*). *C* = 8 rats and *B* = 11 rats. Results are presented as individual values and medians and were analyzed by the Mann–Whitney U-test

In the forced swimming test (FST), there was no statistical significant difference in the time of struggling, swimming or floating between controls and buserelin-treated rats (Fig. 7). The numbers of diving were recorded, but no difference between the groups was detected. These parameters were analyzed and presented for the entire 0–10-min period studied. When the data were analyzed in periods of 0–5 min and 5–10 min, the results did not differ from the presented results (data not shown).

## Discussion

The main findings in the present study of a rat model with buserelin-induced enteric neuropathy were that the gut microbiota composition, intestinal permeability, and stress behavior responses are well preserved despite a neuronal loss of up to 50 % with imbalance in the CRF immunoreactivity. In colon, buserelin treatment increased the relative numbers of submucosal and myenteric CRF-IR neurons, increased the relative number of NOS-IR submucosal neurons also IR to CRF, and tended to increase both the percentage of NOS- and VIP-IR myenteric neurons also IR to CRF. As buserelin did not affect ACTH- and CRF levels in plasma, the HPA axis was not altered in the model. Although the diversity of the dominating taxa of the gut microbiota was unaffected by buserelin, assessed by calculation of richness and diversity, the amount of *Enterobacteriaceae* was lowered in colonic tissue. Intestinal permeability was not affected during basal conditions in either group, but the carbachol-induced increase of ileum permeability in controls was totally attenuated in buserelin-treated rats. Buserelin treatment did not affect stress behavior.

The mechanisms behind the enteric neuronal cell loss can only be speculated on. Raised serum levels of estradiol after buserelin treatments, with secondary effects on vaginal smears and the uterine muscle layer [8], confirm that there are elevated levels of FSH- or LH secretion during the dose regimen of the rat model [5]. Intermittent stimulation of LH in cell culture led to reduced survival of rat enteric neurons, whereas GnRH did not affect the neuronal survival [20]. The enteric neuronal loss in rat was most pronounced in colon, where also the relative number of enteric neurons IR to LH receptors was decreased after buserelin treatment [5]. As no inflammation was present in the bowel wall or in the circulation [5, 8], and the relative number of neurons IR to activated caspase-3 was increased prior to neuron reduction [5], apoptosis is assumed to be involved in the neuronal loss. For comparison, stimulation of the LH receptors on human ovarian granulosa cells leads to increased apoptosis. The mechanisms involved may be mediated through cAMP, since cAMP is able to induce apoptosis in a variety of cell types, and its concentration was increased after receptor stimulation [21]. In analogy, a down-regulation of LH receptors is accompanied by decreased apoptosis [22]. Taking into consideration all studies on enteric neuropathy and GnRH in man and rat, the buserelin-induced GI damage is postulated to be a degenerative and/or inflammatory enteric neuropathy driven by apoptosis; other pathological changes appear to be secondary to the neuropathy [2, 3, 5, 8, 23].

Adult neurons require ability to adapt, referred to as plasticity, to maintain their primary functions when exposed to pathophysiological situations. When several neurotransmitters were studied, all were equally distributed in buserelin-treated rats and controls [8]. A reduced number of neurons along the GI tract would hypothetically lead to GI dysfunction. However, GI transit time, galactose absorption, and body weight were un-affected, and the only significant functional change after buserelin treatment was an increased fecal fat content, suggesting fat maldigestion [8]. A huge reserve capacity of ENS was suggested in the former study with preserved gut function after enteric neuronal loss of up to 50 % [8], and is confirmed in the present study. CRF receptor 1 is described on myenteric neurons in rat and man [14, 15, 17], and CRF increases both secretion and motor activity of the GI tract [10, 17]. Thus, the present finding of relative increase in CRF-IR neurons in colon, the only affected neurotransmitter observed [8], could theoretically counteract a reduced GI function. Bacterial overgrowth is often related to anatomical or motility disturbances of the GI tract, but is a late sign of dysfunction [24]. Our results of unaffected bacterial amount or diversity, and intact total GI transit time [8], indicate that the motility patterns are well preserved with only half of its neurons intact. The reduced level of *Enterobacteriaceae* in colon may be the first sign of disturbed balance in enteric neurons and CRF immunoreactivity, alterations which were most pronounced in colon. The *Enterobacteriaceae* family is associated with inflammation [25] as is also true for CRF [14]. The inflammatory process in ulcerative colitis is supposed to be modulated by peripheral CRF receptors [16]. The expression of TLR-4 on epithelial cells was increased by CRF receptor stimulation in vitro and after sham stress, which decreased the established tolerance of the epithelial barrier from Gram negative bacteria and their products, e.g. LPS [26]. CRF receptors have also been found in *Escherichia coli*, which belongs to the *Enterobacteriaceae* family [27]. Hypothetically, the decrease of these bacteria in colon may be a protection mechanism due to increased CRF levels, to counteract effects by pro-inflammatory factors, and to prevent an inflammatory response and retain intact intestinal permeability [16, 19, 26]. Causes or consequences of modifications in microbiota must be further studied in prospective studies.

Nitric oxide and, in particular VIP, have been established to induce intestinal relaxation and secretion and to take part in neuronal survival [28, 29]. As up-regulation of VIP is seen in several pathophysiological situations, enteric neurons with the ability to increase their VIP expression have been suggested to be better protected against neuronal injuries [30]. In the present study where the rats were euthanized 2 weeks after the fourth treatment session, no increases of relative numbers of NOS- or VIP-IR neurons were found, as in the former study where the rats were euthanized immediately afterwards [6]. The neuronal tissue damage might have subsided 2 weeks later. The increased percentage of NOS- and VIP containing neurons also IR to CRF in the present study could possibly hamper the protective effect of NOS and VIP, since CRF in previous studies has been shown to counteract the protective effect of VIP [19]. In this context, it may be noted that no direct association between intestinal CRF levels and gut dysfunction in intensive care unit patients was found [31].

Cholinergic stimulation by carbachol mimics stimulation during a meal and has been shown to increase the permeability in rat epithelium [17], in accordance to our findings of increased ileum uptake of mannitol, FITC-dextran, and BSA in controls. As nerve-mast cell interactions are important in the rat barrier function [17], the inability of buserelin-treated rats to respond to carbachol might reflect the reduced neuronal number in ileum. Other components also may be involved. In jejunal submucosal tissues from pig, the barrier function was regulated by muscarinic receptors on epithelial cells, independently of ENS [32]. Whether the present functional loss observed is due to lack of submucosal or myenteric neurons, or both, remains unclear as we used un-separated, whole-wall mounts in the Ussing chamber studies.

There is a well-described interaction between CRF, mast cells, and enteric nerves in rodents [33]. In experimental studies, CRF receptor 1 situated on mast cells has been suggested as at least partly responsible for the increase of mucus secretion, para- and transcellular permeability, and short-circuit currents induced by acute and chronic stress [10, 17, 33]. The effects are abolished in mast cell deficient rodents or blocked by CRF receptor 1 antagonists [10]. As the percentages of CRF-IR neurons in ileum in the present study were equal in both groups, CRF was not involved in the carbachol-induced permeability. A concomitant inflammation with increased number of mast cells, which was not seen in our rat model [5], could theoretically have reinforced the effects of a reduced number of enteric neurons.

In man, high intestinal integrity is important for health, and enhanced intestinal permeability influences the development of several diseases, e.g. irritable bowel syndrome (IBS) and depression [34]. Visceral hypersensitivity is characteristic for patients with IBS, and stress is shown to exacerbate IBS symptoms [9]. This visceral sensitivity has been supposed to be mediated by brain CRF signaling, but more recent research suggest an equal contribution of the peripheral CRF signaling [10, 14]. Both increased permeability and visceral hypersensitivity seem to be mediated via CRF stimulation of mast cells [35, 36], which are found in close proximity to colonic neurons in IBS patients [37]. An exaggeration of the HPA axis has been shown in IBS patients, resulting in hyper-responsiveness to CRF injections with enhanced colonic motility [38]. The microbiota composition affects the barrier function, and probiotic treatment attenuated the HPA axis to stress and blocked the stress-induced hyper-permeability [39]. Taken together, although IBS-like symptoms sometimes appear after GnRH treatment [7], our rat model does not represent an IBS model since neither the gut microbiota, mast cell account [5], basal intestinal permeability or HPA axis were influenced. Clinical examinations during stimulation by carbachol could be useful methods to differ between IBS and enteric neuropathy. In conclusion, this and our former study [8] suggest that patients with diffuse GI complaints may have enteric neuropathy although no organic changes may be found in standardized clinical examinations.

The FST *per se* induces increased ACTH secretion as a response to swimming [40]. Local injection of CRF to the nucleus accumbens in rats during the EPMT and FST increased anxiety-like and depression-like behaviors [41]. In a rat model of gastric irritation with central elevated levels of CRF immunoreactivity and ACTH, the CRF-1 receptor antagonist antalarmin has been shown to reverse the depression-like behavior in FST [13]. These results suggest endogenous CRF as a key player in behavior responses, and both peripheral and central mechanisms may be involved. However, the stress behavior in the present study was not affected by the increased CRF immunoreactivity in colon.

Altered central processing, early life events, and cognitive and psychosocial factors are considered necessary prerequisites to develop functional gastrointestinal bowel diseases (FGBD) with comorbidity of affective disorders [42]. Significant differences in the neural processing of pain between IBS patients and controls further underline the importance of central factors in the pathophysiology of visceral hypersensitivity [43], and moderate enteric disease *per se* may not influence the development of depression.

Future studies have to explore the mechanisms why the buserelin-induced effects with a relative increase of CRF immunoreactivity and lowered amount of *Enterobacteriaceae* are most pronounced in colon. The increased CRF immunoreactivity in submucosal neurons seems to be due to a protection in CRF-IR neurons to death, whereas the increased CRF immunoreactivity in myenteric ganglia seems to be due to de-novo CRF gene activation. Why CRF is affected differently compared with other subpopulations of neuropeptides has to be examined [8]. Also, the expression of CRF receptors would be of interest to examine, but the immunocytochemistry with available antibodies has failed so far.

## Conclusion

A rat model of GnRH analog-induced enteric neuronal loss, without concomitant inflammation, has a well preserved gut function although it renders an increased relative number of CRF-IR enteric neurons, lowered levels of *Enterobacteriaceae* in colon, and an inability of ileum to respond to carbachol with increased permeability. When several functions of the GI tract have been examined, the only significant GI dysfunctions measured are increased fecal fat content [8] and an inability of carbachol to increase ileum permeability. Both these dysfunctions reflect impaired small intestinal functions, and increased relative CRF immunoreactivity and lowered amount of *Enterobacteriaceae* were only identified in colon. Thus, GI dysfunction in buserelin-induced neuropathy in rat is not related to altered gut microbiota or CRF immunoreactivity.

## Methods

### Animals

Female Sprague–Dawley rats (n = 60, 7 weeks of age, 170–180 g), purchased from Charles River, Sulzfeld, Germany, were used. The rats were allowed to acclimatize to the climate- and light-controlled animal facility for at least 5 days prior to experimentation. Water and standard rat chow (4 % fat/g) (Lactamin R36, Stockholm, Sweden) were supplied at all times, except when withdrawn during experimental tests. The experimental design was approved by the Animal Ethics Committee, Lund and Malmö, Sweden (M350-12, date of approval 14.11.12). Animals were used in accordance with the European Communities Council Directive (2010/63/EU) and the Swedish Animal Welfare Act (SFS 1988:534).

### Study design

Rats (n = 36) were given 20 μg of the GnRH analog buserelin (Suprefact^®^ [1 mg/ml], Sanofi-Aventis, Bromma, Sweden) (dissolved in 0.2 ml saline) subcutaneously, once daily for 5 days, followed by 3 weeks of recovery, representing one session of treatment (for details see Sand et al. [5]). All rats received four treatment sessions. The dosage and administration of buserelin are based on previous studies showing reliable physiological effects in terms of uterine hypertrophy, synchronization of the hormonal cycle, and elevated estradiol levels after four treatment sessions, without any adverse effects [5, 8]. Control rats (n = 24) received saline injections (0.2 ml). The rats were weighed prior to inclusion in the study, and weekly in the morning during the study, using electronic scales. As histological analyses in previous studies were performed immediately after the fourth treatment session, with very modest effects on GI structure except neuropathy and ganglioneuritis [5, 23], we wanted to perform the present analyses a few weeks later to get further information about the chronic course.

Two weeks after the fourth treatment session, 12 buserelin-treated and eight control rats (26 weeks of age) were anaesthetized with chloral hydrate (300 mg/kg) in the morning and thereafter euthanized through aorta puncture. Blood was collected and plasma was analyzed for concentrations of ACTH and CRF. Tissue samples from the fundus, distal ileum, proximal colon, and uterus were collected and rinsed in saline before being fixed and processed for cryo/paraffin sectioning. The total number of enteric neurons was assessed, as well as the relative numbers of neurons IR to CRF, NOS, and VIP. Tissue samples of ileum and colon were collected for analyses of intestinal microbiota and placed on dry ice directly after removal from the rat, and stored at −80 °C, before being analyzed by Terminal Restriction Fragment Length Polymorphism (T-RFLP), next generation sequencing, and quantitative polymerase chain reaction (qPCR).

Twelve buserelin-treated and eight control rats (26–28 weeks of age) were euthanized during a 2-week period, with start 10 days after the fourth treatment session, for examination of intestinal permeability in Ussing chambers.

Twelve buserelin-treated and eight control rats (27–28 weeks of age) were examined by EPMT at day 10 and 11, and by FST at day 17 and 18, after the fourth treatment session, to assess anxiety-like behavior and depression-like activity, respectively, with ability for the rats to rest in-between.

### Blood preparation and plasma analyses

Blood samples were collected in lithium heparin tubes (BD Microtainer, New Jersey, USA) through aorta puncture. Blood was centrifuged at 3000 rcf (1.12 × R × (RPM/1000)^2^) for 5 min and plasma was stored at −20 °C.

The levels of ACTH in plasma were analyzed in duplicate with a competitive inhibition enzyme-linked immunosorbent assay (ELISA) kit (CEA836Ra lot no L40505077, Cloud-Clone Corp., Nordic Diagnostica AB, Billdal, Sweden) according to the manufacturer’s manual. Standards and rat heparin plasma, diluted 1:2 in 0.01 M phosphate-buffered saline (PBS), pH 7.2, were incubated in a plate which was pre-coated with a monoclonal anti-ACTH antibody. The unbound conjugate was washed off and avidin-conjugated horseradish peroxidase (HRP) was added. The wells were incubated with 3,3′, 5,5′-Tetramethylbenzidine (TMB) for HRP enzyme, and the product of the enzyme-substrate reaction formed a blue-colored complex. The reaction was stopped and the absorbance was measured at the optic density of 450 nm. The amount of bound HRP conjugate was reversed proportional to the concentration of ACTH in the samples, and the ACTH concentration in each sample was interpolated from the standard curve.

CRF levels in plasma were analyzed with a commercial ELISA kit (RSCYK131R lot no 2130719, Biovendor, Awakura, Fujinomiya-Shi Shizuoka, Japan) based on a sandwich enzyme immunoassay. The plate was coated with antibodies against mouse/rat CRF, and standards (0.0, 0.039, 0.078, 0.313, 0.625, and 1.25 ng/ml) and plasma (50 µl/well) were added in duplicate for the first step of immunoreaction. Second, a biotinylated rabbit anti-mouse/rat antibody was added to form a labeled antigen–antibody complex, and third, streptavidin-HRP was added. Substrate was then added to the wells and after a proper incubation time, the reaction was stopped with a sulfuric acid solution. The color change was measured at 450 nm and the sample concentration was interpolated from the standard curve.

### Morphometric and neuronal survival experiments

#### Tissue preparation

A total of 20 female rats (12 buserelin-treated and eight controls) were used. The fundus/corpus region of the stomach, ileum (with start 8 cm proximally to cecum and continuing in proximal direction), colon (with start 2 cm distally to cecum and continuing in distal direction), and uteri were opened and flattened on filter paper. One portion of each gut segment was fixed in Stefanini’s fixative (a mixture of 2 % formaldehyde and 0.2 % picric acid in phosphate buffer, pH 7.2) for 22 h at 4 °C. The other portion of gut segments and uteri were fixed in 4 % paraformaldehyde in 0.1 M phosphate buffer for 22 h at 4 °C. Stefanini-fixed, whole-wall specimens were rinsed in Tyrode’s solution containing 10 % sucrose three times before being oriented and mounted for cross- and longitudinal sectioning in Tissue-Tek (Sakura, Histolab, Göteborg, Sweden), frozen on dry ice, and sectioned (10 μm). Paraformaldehyde-fixed, whole-wall specimens were dehydrated in ethanol, cleared in xylene, oriented for cross- and longitudinal sectioning, embedded in paraffin, and sectioned (5 µm). Sections were processed for immunocytochemistry and histochemistry.

#### Histochemistry

Automated measurements of wall layer thickness were performed on scanned, deparaffinized, hydrated, and hematoxylin-eosin-stained paraffin sections from the uterus by using a computerized, image analyzing system (Imagescope, Aperio ScanScope GL SS5082, Vista, CA, USA). The myometrial thickness was measured using a binary cursor; mean values of 6–10 representative measurements were calculated from each rat.

#### Immunocytochemistry

Neuronal survival and enteric neurons IR to CRF (fundus, distal ileum, and proximal colon), NOS (proximal colon), and VIP (proximal colon) were studied as described in detail previously [5, 15]. NOS and VIP were only analyzed in colon, since the previous study showed most prominent effects on NOS- and VIP immunoreactivity in colon [5]. Briefly, to study neuronal survival, monoclonal mouse antibodies against the biotinylated human neural protein (HuC/D; dilution 1:400; code no A-2127, Life Technologies, Stockholm, Sweden) were used as a general neuronal marker and visualized with a VECTASTAIN ABC kit containing HRP and 3,3′-diaminobenzidine tetrahydrochloride (DAB) (Vector Laboratories, Inc., CA, USA). The relative number of CRF-IR neurons was examined by CRF antibodies raised in rabbit (dilution 1:1600–1:3200; code no C-5348, Sigma-Aldrich, St Louis, MO, USA) in combination with antibodies against HuC/D visualized with a mixture of DyLight TM 488-conjugated goat anti-mouse IgG serum and Alexa Fluor TM 594-conjugated donkey anti-rabbit IgG serum. The relative numbers of CRF-IR neurons also IR to NOS or VIP were studied by CRF antibody in combination with NOS antibodies raised in sheep (dilution 1:3200; code no 1529, Chemicon international, Billerica, MA, USA) or monoclonal VIP antibodies raised in mouse (dilution 1:5000; code no 9535-0504, Biogenesis Ltd, Poole, UK). These two combinations were visualized by exposure to DyLight TM 488-conjugated donkey anti-goat IgG serum and Alexa Fluor TM 594-conjugated donkey anti-rabbit IgG serum (dilution 1:1000) or to DyLight TM 488-conjugated goat anti-mouse IgG serum and Alexa Fluor TM 594-conjugated donkey anti-rabbit IgG serum (dilution 1:1000), respectively. All secondary antibodies were from Jackson ImmunoResearch Laboratories, Inc., Novakemi AB, Handen, Sweden. Synthetic antigens for testing the specificity of antibodies against HuC/D are not commercially available. Thus, omission of the primary antibodies was used as controls. Regarding the specificity of NOS and VIP, absorption controls were performed by adding an excess amount of antigen (10–100 µg of synthetic peptide diluted in antiserum) before exposure.

The number of HuC/D neurons in colon was counted in scanned, cross- and longitudinally cut, whole-wall sections and evaluated in a total length of at least 30 mm, cut at 6–9 different depths per region and rat. Neuronal survival is expressed as numbers of HuC/D-IR submucosal or myenteric neurons per mm region length. At least 150 submucosal and 250 myenteric HuC/D-IR neurons were estimated for simultaneous co-localization of CRF from each region and rat. At least 30 mm section lengths were analyzed for neuron immunoreactivity to NOS and VIP in addition to CRF. The results on the subpopulations are expressed as the percentage of HuC/D-, NOS- or VIP-IR neurons also IR to CRF.

### Microbial analyses

#### DNA extraction

Whole-wall tissue samples from 20 female rats (12 buserelin-treated and eight controls) were used, since the bacteria content in the mucosa is more representative for the microbiota than feces. DNA from distal ileum and proximal colonic tissue (0.03–0.06 g) was isolated and purified in EZ1 Advanced XL (EZ1 DNA Tissue kit and Bacteria card, Qiagen, Hilden, Germany). Samples were incubated with 380 µl Buffer G2 and 30 µl Proteinase K (Qiagen) and 12 glass beads (2 mm in diameter) at 56 °C until totally lysed (for approximately 4 h). The lysates were shaken at 4 °C for 45 min to further disintegrate the bacterial cell walls. The lysates were spin down shortly and 200 µl was used for extraction in the EZ1 Advanced XL.

#### Microbial diversity

To assess the microbial diversity, T-RFLP was applied. The *16SrRNA* genes were amplified with the universal forward primer FAM-ENV1 (5′-AGA GTT TGA TII TGG CTC AG-3′), fluorescently labeled with carboxyfluorescein (6-FAM) at the 5′ end, and the reverse primer ENV2 (5′-CGG ITA CCT TGT TAC GAC TT-3′) (I = inosine), which anneal by 8–27 base pairs (bp) and 1511–1492 bp, respectively. The PCR reaction mixture contained 0.4 µM FAM-ENV1 primer, 0.2 µM ENV2 primer (Eurofins MWG, Ebersberg, Germany), 0.2 mM of each deoxyribonucleotide triphosphate (Qiagen), 2.5 µl of 10× TopTaq PCR reaction buffer (containing 15 mM MgCl_2_) (Qiagen), 0.625 units TopTaq DNA Polymerase (Qiagen), 2.5 µl of 10× CoralLoad (Qiagen), and 2.5–6 µl of template, in a final volume of 25 µl. Amplification was made in an Eppendorf Mastercycler (Hamburg, Germany) using the following program: one cycle at 94 °C for 3 min, followed by 30 cycles; denaturation at 94 °C for 1 min, annealing at 50 °C for 45 s, and extension 72 °C for 2 min, with an additional extension at 72 °C for 10 min in the end. PCR products were verified on 1.5 % agarose gel with GelRed nucleic acid gel stain (Biotium, Hayward, CA, USA). Products from three PCR reactions were pooled, purified, and concentrated by MinElute PCR Purification Kit (Qiagen) according to the manufacturer’s protocol. The DNA was eluted in 20 µl of RNAse-free water (Qiagen) and the concentration was measured by NanoDrop ND-1000 Spectrophotometer (NanoDrop Technologies, Wilmington, DE, USA) using 1 µl purified PCR product. T-RFLP analysis was performed with *Msp*I and electropherograms were analyzed with GeneMapper^®^ version 4.1 (Applied Biosystems, Foster city, CA, USA) as described elsewhere [44]. Thresholds for internal standard and terminal restriction fragments (T-RFs) were set to 18 and 40 fluorescence units, respectively.

#### Next generation sequencing

Partial *16S rRNA* gene sequences of the V3 region were amplified from extracted DNA of rat colon using Phusion High-Fidelity DNA Polymerase (F-530S, Thermo Scientific, MA, USA). For each community a different forward primer, with a 10–12 bp unique barcode, was used to identify the different samples. Furthermore, the forward primers contained the A-adapter and the universal reverse primer 581R included the trP1-adapter, both needed for Ion Torrent sequencing. The complete list of primers used in this study is reported in Additional file 3: Table S1. The PCR conditions used were 98 °C for 30 s, followed by 27 cycles; denaturation at 98 °C for 15 s and annealing at 72 °C for 30 s followed by extension at 72 °C for 5 min, and then cooled to 4 °C. PCR products were purified by electrophoretic separation on a 1.5 % agarose gel and the use of MinElute Gel extraction kit (Qiagen). DNA concentrations were determined with Qubit^®^ high sensitivity assay (Life Technologies) and the amount was adjusted so an equimolar mixture of all the different libraries was obtained. Sequencing was performed on a 318 chip using the Ion OneTouch™ 200 Template Kit v2 DL (Life Technologies). After sequencing, the individual sequence reads were processed in the CLC BIO genomic workbench version 6 (CLC Bio, Aarhus, Denmark) to remove low quality and polyclonal sequences. Approximately 40,300 good quality reads of 110–180 bp were obtained from each sample and further processed in the Ribosomal Database Project. The domain Bacteria was used as reference when calculating the relative abundance of phyla and families.

#### Quantitative PCR

The total amount of bacteria and the bacterial abundance of *Clostridium leptum, Enterobacteriaceae*, and *Bacteroides fragilis* were estimated in tissue from ileum and colon, using separate quantitative PCR assays according to Karlsson et al. [45]. Primers used for the qPCR assays have been published previously [46–48]. The detection limit for *C.leptum* and *Enterobacteriaceae* was 10^2^ gene copies/reactions, whereas the limit for total bacteria and *B.fragilis* was 10^3^ gene copies/reactions. For standard curves, tenfold dilution series of the target DNA were made in EB buffer (Qiagen). Number of bacteria was expressed as log_10_*16S rRNA* gene copies/g ileum or colon tissue.

#### Calculations

Microbial diversity was estimated by calculation of richness (number of T-RFs) and Shannon and Simpson diversity indices as described by Karlsson et al. [45], with the exception that T-RFs within 40–580 bp were included in the T-RFLP profile analysis and calculation. The diversity indices take into accountability both richness and evenness when considering the relative abundance of bacterial groups. Both indices are commonly used to assess microbial diversity [49]. Samples under the detection limit in the qPCR runs were set to the highest possible detection limit for statistical analysis.

### Ussing chamber experiments

#### Procedure

A total of 20 female rats (280–320 g; 12 buserelin-treated and eight controls) were anaesthetized with chloral hydrate (300 mg/kg) in the morning. The distal half of the small intestine and the proximal half of the colon was dissected, rinsed, and immersed in room-tempered modified Krebs buffer (0.1 M NaCl/3 mM CaCl_2_/5.5 mM KCl/14 mM KH_2_PO_4_/29 mM NaHCO_3_/5.7 mM Na-pyruvate/7 mM Na-fumarate/5.7 mM Na-glutamate/13.4 mM glucose/pH 7.4), oxygenated with carbogen (95/5 %, O_2_/CO_2_). The intestine was then cut in 3 cm sections, opened along the anti-mesenteric border, and the whole tissue was mounted in the Ussing diffusion chambers (exposed intestinal area of 1.78 cm^2^) in accordance with previous description [50, 51]. Each half-cell of the Ussing chambers were filled with 5 ml of Krebs buffer, kept at 37 °C, and connected to carbogen supply. All intestinal segments were mounted in the chambers within 30 min after dissection and were considered viable for 2 h [52].

At start of the experiment, the buffer was exchanged to a fresh Krebs buffer in the serosal half-cell, and to a marker-containing buffer in the mucosal half-cell. This consisted of fresh Krebs buffer, in which glucose was exchanged with mannitol (13.4 mM), containing the marker molecules d-(1-^14^C)-mannitol (182 Da, chamber concentration: 2.9 kBq/mL, specific activity of the isotope: 2.035 GBq/mmol; PerkinElmer, Massachusetts, USA), FITC-dextran (4 000 Da, chamber concentration: 1 mg/mL; TdB Consultancy AB, Uppsala, Sweden), and BSA (66 000 Da, chamber concentration: 25 mg/mL; A-4503, Sigma-Aldrich). One section of ileum and colon were exposed to the marker solution, and in addition, one part of the ileum was supplemented with the acetylcholine receptor agonist carbamoylcholine, also called carbachol (Carbachol^®^, chamber concentration: 10 mM, Sigma-Aldrich) in the serosal buffer. Serosal samples of 1 ml were collected every 20 min during 120 min and replaced with the same volume by fresh buffer.

#### Analyses

The amount of radio-labeled mannitol in the serosal samples was measured in a beta counter (Scint TriCarb Liquid Scintillation Analyzer 2100TR, PerkinElmer), after mixing 0.5 ml of the serosal sample with 5 ml liquid scintillation cocktail (Optiphase ‘Highsafe’ 2, Perkin Elmer). FITC-dextran was quantified by fluorescence spectrophotometry (CytoFlour™ 2300, Millipore Corp., Bedford, MA, USA) at an excitation wavelength of 480 nm and an emission wavelength of 520 nm. FITC-dextran dissolved in modified Krebs buffer was used as a standard. Quantification of BSA was carried out by electro immunoassay in agarose gel [53] with an antisera specific to BSA (B-1520 Rabbit Anti-Bovine Albumin, Sigma-Aldrich), using purified BSA (A-7639, Sigma-Aldrich) as the standard.

#### Calculations

The passage of d-(1-^14^C)-mannitol, FITC-dextran and BSA over the intestinal segments was determined as the percentage passage from mucosal to serosal half-cell at indicated time-points.

### Stress behavior tests

A total of 20 female rats (12 buserelin-treated and eight controls) were analyzed by stress behavior tests.

#### Elevated plus maze test

The EPMT has been developed to assess anxiety-like behavior [54]. The apparatus consisted of two open arms (50 × 10 cm^2^) surrounded by a 1-cm-high Plexiglass and two closed arms (50 × 10 × 38 cm^3^) that emerge from a central platform. The apparatus was made from dark grey PVC and the arms were elevated 73 cm above the floor. A white incandescent bulb provided a light intensity of 300 lux over the open arms and of 20 lux over the closed arms. At the beginning of each trial, the rat was placed on the central platform facing an open arm. The floor of the maze was cleaned with 10 % ethanol before each trial. The rat behavior was videotaped over 10 min (divided in one initial and one latest 5-min session), and a trained observer blinded to the treatment groups scored the parameters from the videotape. The behavioral parameters scored were: the numbers of total entries; the numbers of entries into closed and open arms (an entry was counted when both forepaws were placed on the respective arm); the time spent in the closed arms, open arms, and in the center platform; and the numbers of entries into the outer part of the open arms and the time spent their. The numbers of open arm head dips (standing on the open arm, leaning the head over the Plexiglass edge) and protected arm head dips (standing with the body in the protected arms, leaning the head over the Plexiglass edge) were investigated.

#### Forced swimming test

The procedure of FST was a modification of that described by Porsolt et al. [55]. Animals were placed in individual glass cylinders (diameter: 25 cm, height: 60 cm) containing water (height: 40 cm, temperature: 25 °C). One 10-min session was conducted. The duration in seconds of struggling, swimming, and floating were manually measured during the initial and latest 5-min period of the test session by an experimenter unaware of the treatment groups. The numbers of diving were recorded. Struggling was defined as strong movement of all four limbs with the front paws breaking the water surface or scratching the glass cylinder wall. Swimming was defined as movements of 2–4 limbs, swimming around in the tank or diving. Floating occurred when rats remained immobile with only occasional slight movements to keep the body balance and the nose above the water.

### Statistical analyses

Results are presented as median (interquartile range [IQR]), except for weight which is expressed as mean ± standard error of the mean (SEM). Statistical analyses were performed in GraphPad Prism 6 (GraphPad Software, Inc., La Jolla, CA, USA) using the Student’s *t* test for the normally distributed weight variable and the Mann–Whitney U-test for the other variables due to the low number of animals. P < 0.05 was considered statistically significant.

## Additional files

10.1186/s13104-015-1800-x Numbers of neurons in submucosal (SG) and myenteric ganglia (MG) per mm length in colon from rats treated with four sessions of saline (controls, C) or buserelin (B). Rats were euthanized 2 weeks after the fourth treatment session. Neuronal counting was performed on longitudinally cut, whole-wall sections. Rats subjected to four sessions of buserelin treatment showed an equal number of submucosal neurons immunoreactive to corticotropin-releasing factor (CRF) in colon compared with controls. The number of myenteric neurons immunoreactive to CRF in colon was increased after buserelin treatment compared with controls (p < 0.05). C = 6 rats and B = 6 rats. Results are presented as individual values and medians and were analyzed by the Mann–Whitney U-test. Statistical significance is indicated by * = p < 0.05.
10.1186/s13104-015-1800-x Microbial diversity in rat tissue from ileum and colon.
10.1186/s13104-015-1800-x List of primers used in the present study.

## Abbreviations

ACTH: adrenocorticotropic hormone
BSA: bovine serum albumine
CRF: corticotropin-releasing factor
ENS: enteric nervous system
EPMT: elevated plus maze test
FITC: fluorescein isothiocyanate
FSH: follicle-stimulating hormone
FST: forced swimming test
GnRH: gonadotropin-releasing hormone
HPA: hypothalamic–pituitary–adrenal
HuC/D: human neuronal protein
IBS: irritable bowel syndrome
IR: immunoreactive
LH: luteinizing hormone
MG: myenteric ganglia
NOS: nitric oxide synthase
SG: Submucosal ganglia
T-RFLP: terminal restriction fragment length polymorphism
VIP: vasoactive intestinal peptide
