# Supplementary figures and images for: Buserelin treatment to rats causes enteric neurodegeneration with moderate effects on CRF-immunoreactive neurons and Enterobacteriaceae in colon, and in acetylcholine-mediated permeability in ileum
Source: BMC Res Notes. 2015 Dec 28;8:824. doi: 10.1186/s13104-015-1800-x (PMC4693429; doi:10.1186/s13104-015-1800-x)

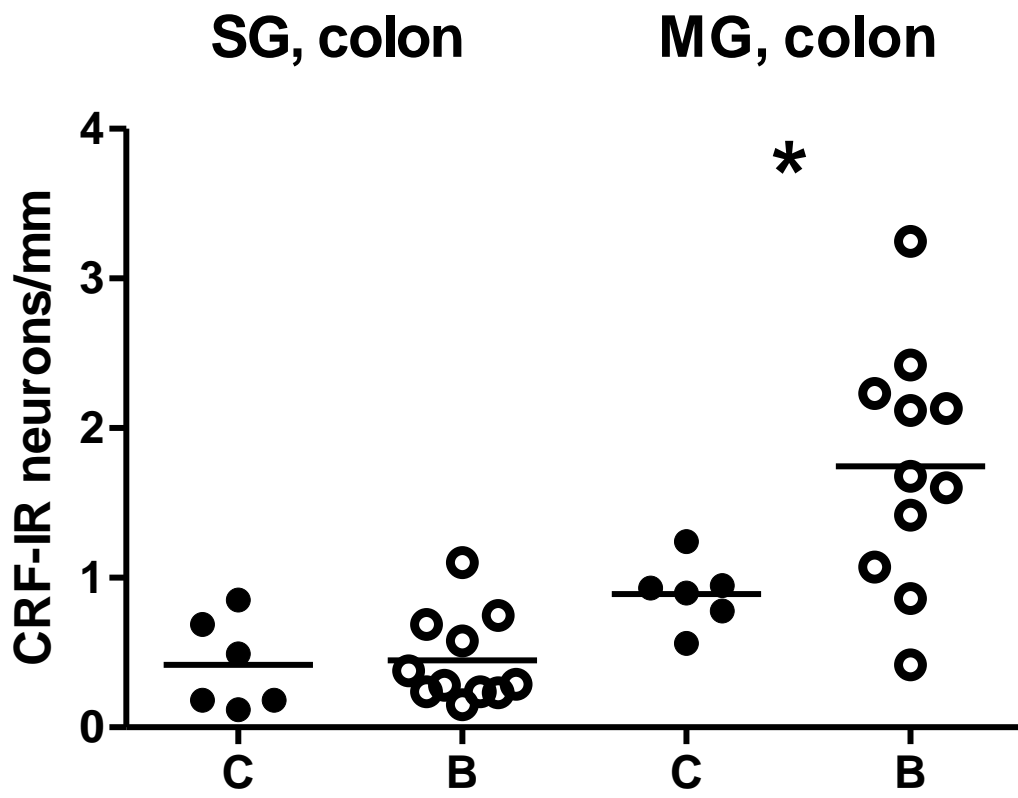

Supplement: Supplementary file 1 — 10.1186/s13104-015-1800-x Numbers of neurons in submucosal (SG) and myenteric ganglia (MG) per mm length in colon from rats treated with four sessions of saline (controls, C) or buserelin (B). Rats were euthanized 2 weeks after the fourth treatment session. Neuronal counting was performed on longitudinally cut, whole-wall sections. Rats subjected to four sessions of buserelin treatment showed an equal number of submucosal neurons immunoreactive to corticotropin-releasing factor (CRF) in colon compared with controls. The number of myenteric neurons immunoreactive to CRF in colon was increased after buserelin treatment compared with controls (p < 0.05). C = 6 rats and B = 6 rats. Results are presented as individual values and medians and were analyzed by the Mann–Whitney U-test. Statistical significance is indicated by * = p < 0.05. [file 13104_2015_1800_MOESM1_ESM.pdf]
