# Supplementary material for: Buserelin treatment to rats causes enteric neurodegeneration with moderate effects on CRF-immunoreactive neurons and Enterobacteriaceae in colon, and in acetylcholine-mediated permeability in ileum
Source: BMC Res Notes. 2015 Dec 28;8:824. doi: 10.1186/s13104-015-1800-x (PMC4693429; doi:10.1186/s13104-015-1800-x)
Supplement: Supplementary file 2 — 10.1186/s13104-015-1800-x Microbial diversity in rat tissue from ileum and colon. [file 13104_2015_1800_MOESM2_ESM.docx]

#### Supplemental Table 2. Microbial diversity in rat tissue from ileum and colon

|  | Saline-treated rats  N = 7 | Buserelin-treated rats  N = 11 | P-value |
| --- | --- | --- | --- |
| No T-RFs, ileum | 14 (7.5–23.7) | 18 (12.25–18.75) | 0.928 |
| Shannon index, ileum | 1.98 (1.57–2.26) | 2.38 (1.60–2.27) | 0.526 |
| Simpson index, ileum | 0.74 (0.71–0.86) | 0.81 (0.76–0.89) | 0.319 |
| No T-RFs, colon | 35 (31.25–40) | 37 (32.5–41.5) | 0.526 |
| Shannon index, colon | 3.27 (2.93–3.38) | 3.21 (3.13–3.33) | 0.928 |
| Simpson index, colon | 0.95 (0.91–0.96) | 0.94 (0.94–0.95) | 0.856 |

Values are given as median (interquartile ranges). T-RF = Terminal restriction fragments.

Shannon and Simpson indices were used to calculate gut microbial diversity.
