# Supplementary material for: Buserelin treatment to rats causes enteric neurodegeneration with moderate effects on CRF-immunoreactive neurons and Enterobacteriaceae in colon, and in acetylcholine-mediated permeability in ileum
Source: BMC Res Notes. 2015 Dec 28;8:824. doi: 10.1186/s13104-015-1800-x (PMC4693429; doi:10.1186/s13104-015-1800-x)
Supplement: Supplementary file 3 — 10.1186/s13104-015-1800-x List of primers used in the present study. [file 13104_2015_1800_MOESM3_ESM.docx]

| **Rat no** | **Primer name** | **Sequence 5'-3'** |
| --- | --- | --- |
|  | PBR_P1, 518R | CCTCTCTATGGGCAGTCGGTGATattaccgcggctgctgg |
| C1 | PBU_22 | CCATCTCATCCCTGCGTGTCTCCGACtcag**TTCGAGACGC**gatCCTACGGGAGGCAGCAG |
| C2 | PBU_23 | CCATCTCATCCCTGCGTGTCTCCGACtcag**TGCCACGAAC**gatCCTACGGGAGGCAGCAG |
| C3 | PBU_24 | CCATCTCATCCCTGCGTGTCTCCGACtcag**AACCTCATTC**gatCCTACGGGAGGCAGCAG |
| C4 | PBU_25 | CCATCTCATCCCTGCGTGTCTCCGACtcag**CCTGAGATAC**gatCCTACGGGAGGCAGCAG |
| C5 | PBU_26 | CCATCTCATCCCTGCGTGTCTCCGACtcag**TTACAACCTC**gatCCTACGGGAGGCAGCAG |
| C6 | PBU_27 | CCATCTCATCCCTGCGTGTCTCCGACtcag**AACCATCCGC**gatCCTACGGGAGGCAGCAG |
| C7 | PBU_28 | CCATCTCATCCCTGCGTGTCTCCGACtcag**ATCCGGAATC**gatCCTACGGGAGGCAGCAG |
| B8 | PBU_29 | CCATCTCATCCCTGCGTGTCTCCGACtcag**TCGACCACTC**gatCCTACGGGAGGCAGCAG |
| B9 | PBU_30 | CCATCTCATCCCTGCGTGTCTCCGACtcag**CGAGGTTATC**gatCCTACGGGAGGCAGCAG |
| B10 | PBU_31 | CCATCTCATCCCTGCGTGTCTCCGACtcag**TCCAAGCTGC**gatCCTACGGGAGGCAGCAG |
| B11 | PBU_32 | CCATCTCATCCCTGCGTGTCTCCGACtcag**TCTTACACAC**gatCCTACGGGAGGCAGCAG |
| B12 | PBU_33 | CCATCTCATCCCTGCGTGTCTCCGACtcag**AGCACGAATC**gatCCTACGGGAGGCAGCAG |
| B13 | PBU_34 | CCATCTCATCCCTGCGTGTCTCCGACtcag**TTCAATTGGC**gatCCTACGGGAGGCAGCAG |
| B14 | PBU_35 | CCATCTCATCCCTGCGTGTCTCCGACtcag**CCTACTGGTC**gatCCTACGGGAGGCAGCAG |
| B15 | PBU_36 | CCATCTCATCCCTGCGTGTCTCCGACtcag**TCTGCCTGTC**gatCCTACGGGAGGCAGCAG |
| B16 | PBU_37 | CCATCTCATCCCTGCGTGTCTCCGACtcag**CGATCGGTTC**gatCCTACGGGAGGCAGCAG |
| B17 | PBU_39 | CCATCTCATCCCTGCGTGTCTCCGACtcag**CCTGGTTGTC**gatCCTACGGGAGGCAGCAG |
| B18 | PBU_40 | CCATCTCATCCCTGCGTGTCTCCGACtcag**TTGGCATCTC**gatCCTACGGGAGGCAGCAG |

Supplemental Table 1. List of primers used in the present study
